# Basic aspects for improving the energy conversion efficiency of hetero-junction organic photovoltaic cells

**DOI:** 10.3402/nano.v4i0.21055

**Published:** 2013-07-10

**Authors:** Sou Ryuzaki, Jun Onoe

**Affiliations:** 1 Research Laboratory for Nuclear Reactors, Tokyo Institute of Technology, Tokyo, Japan; 2 Institute of Scientific and Industrial Research, Osaka University, Osaka, Japan; 3 Department of Nuclear Engineering and Research Laboratory for Nuclear Reactors, Tokyo Institute of Technology, Tokyo, Japan

**Keywords:** Crystalline, molecular orientation, external quantum efficiency, intra- and inter-molecular excitons, open-circuit voltage, built-in potential

## Abstract

Hetero-junction organic photovoltaic (OPV) cells consisting of donor (D) and acceptor (A) layers have been regarded as next-generation PV cells, because of their fascinating advantages, such as lightweight, low fabrication cost, resource free, and flexibility, when compared to those of conventional PV cells based on silicon and semiconductor compounds. However, the power conversion efficiency (*η*) of the OPV cells has been still around 8%, though more than 10% efficiency has been required for their practical use. To fully optimize these OPV cells, it is necessary that the low mobility of carriers/excitons in the OPV cells and the open circuit voltage (*V*_OC_), of which origin has not been understood well, should be improved. In this review, we address an improvement of the mobility of carriers/excitons by controlling the crystal structure of a donor layer and address how to increase the *V*_OC_ for zinc octaethylporphyrin [Zn(OEP)]/C_60_ hetero-junction OPV cells [ITO/Zn(OEP)/C_60_/Al]. It was found that crystallization of Zn(OEP) films increases the number of inter-molecular charge transfer (IMCT) excitons and enlarges the mobility of carriers and IMCT excitons, thus significantly improving the external quantum efficiency (EQE) under illumination of the photoabsorption band due to the IMCT excitons. Conversely, charge accumulation of photo-generated carriers in the vicinity of the donor/acceptor (D/A) interface was found to play a key role in determining the *V*_OC_ for the OPV cells.

Organic photovoltaic (OPV) cells consisting of organic donor (D) and acceptor (A) layered films deposited on an indium-tin-oxide (ITO) substrate (see Fig. 1a) have been investigated worldwide as a next-generation PV cell, because the OPV cells have some key advantages compared to conventional PV cells based on silicon and semiconductor compounds (1–6). For example, organic small-molecule and polymer materials, such as metal complexes, fullerenes, and so on, are inherently inexpensive, and the OPV cells are fabricated at low temperatures which results in lower energy consumption and less capital investment than fabrication techniques for Si-based PV cells (2).

**Fig. 1:**
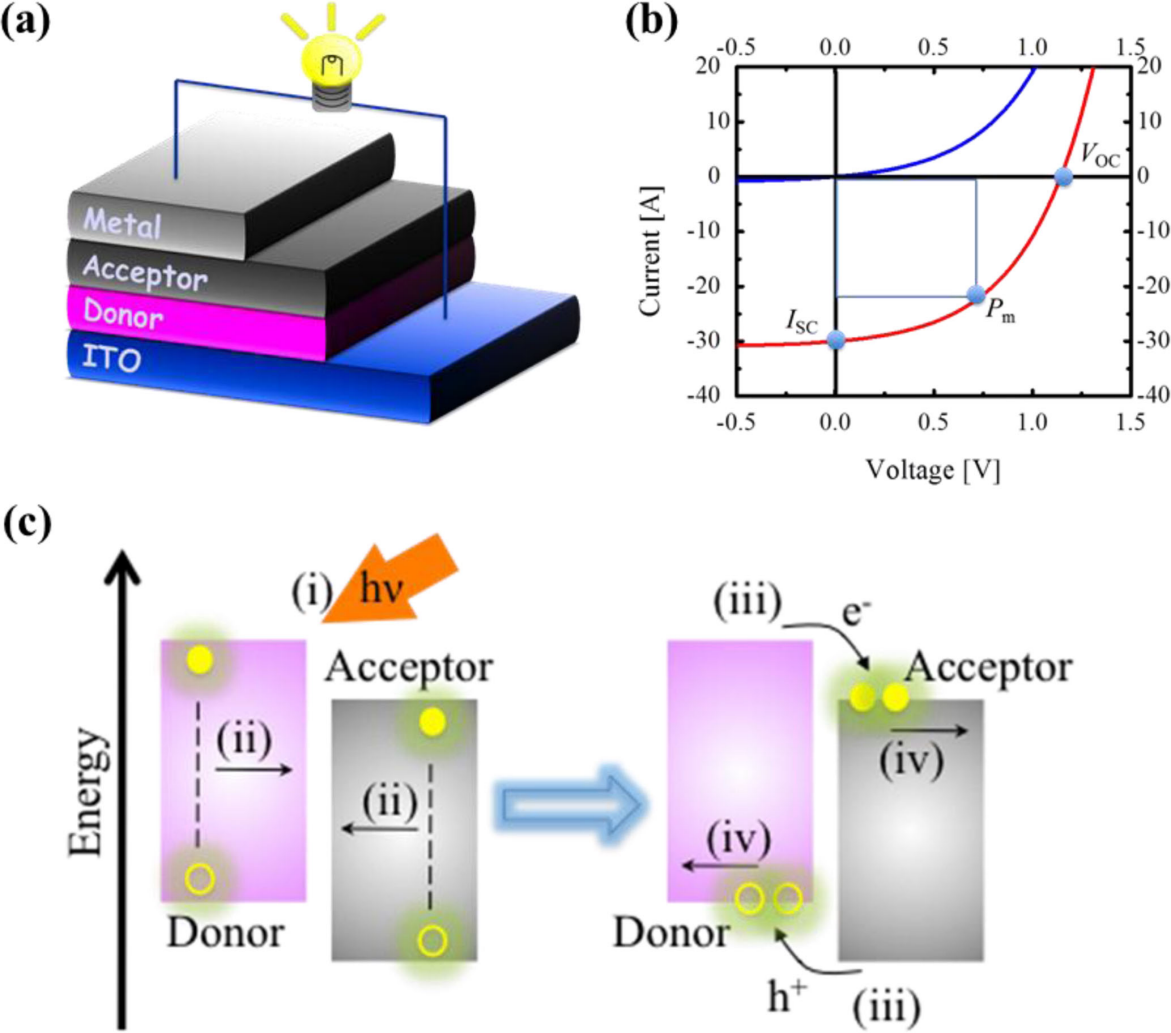
Schematic illustration of a typical hetero-junction OPV cell (a). *I–V* characteristics of photovoltaic cells under dark (blue) and illumination (red) conditions (b). Schematic illustration of photocurrent generation processes for the OPV cells (c).

However, the power conversion efficiency (*η*) of the OPV cells has remained no more than 8% under the standard illuminated condition of AM 1.5 and the threshold efficiency for commercial use is generally agreed to be more than 10% (4–6). The *η* is defined as the ratio of the maximum electrical power (*P*_m_) to the incident optical power (*P*_0_), namely,

1 $$\eta =\frac{P_{\text{m}}}{P_{0}}=\frac{I_{\text{SC}}V_{\text{OC}}\text{FF}}{P_{0}}\times 100$$

2 $$\text{FF}=\frac{P_{\text{m}}}{I_{\text{SC}}V_{\text{OC}}}=\frac{I_{\text{m}}V_{\text{m}}}{I_{\text{SC}}V_{\text{OC}}}$$

Here, *I*_SC_, *V*_OC_, and FF are, respectively, the short-circuit current, the open-circuit voltage, and the fill factor, as shown in Fig. 1b. The *I*_SC_ of the OPV cells is generated by the four processes (efficiencies) shown in Fig. 1c: 1) excitons are generated by light absorption of donor and acceptor films (*η*_A_), 2) the excitons diffuse to the donor/acceptor (D/A) interface (*η*_ED_), 3) carriers (electrons and holes) are generated by the exciton dissociation at the D/A interface (*η*_CG_), and 4) electrons and holes are moved to and collected at the cathode and anode electrodes (*η*_CT_). The OPV cells are often characterized in terms not only of *η* but also of external quantum efficiency (EQE=*η*_A_ *η*_ED_ *η*_CG_ *η*_CT_), because *I*_SC_ is obtained by the integration of EQE obtained at each incident light wavelength (*λ*).

To fully optimize the OPV cells, there are two main issues to be solved: 1) the diffusion length of excitons and carriers is too short (10–30 nm) (7–9), and 2) the open-circuit voltages *V*_OC_ are only in the range of 0.5–1.0 V (10).

We first describe the former issue. Since the diffusion length in crystalline organic films was reported to become longer than in amorphous ones (11), it is important to control the crystal structure, crystalline, and/or molecular orientation of organic films for a longer diffusion length. However, it is hard to obtain an epitaxial growth on an ITO substrate on organic films. Accordingly, the *η* of OPV cells has not been improved significantly, though a higher crystalline organic film is expected to perform a better charge collection at the electrodes in accordance with a low series resistance (8). In this way, the practical influence of the film structural properties on the *I*_SC_ of OPV cells has not been well understood until now.

We next describe the second issue of a lower open-circuit voltage *V*_OC_. Although the *V*_OC_ has been reported to depend on the electronic states in the vicinity of D/A interface such as energy difference (Δ*E*_HL_) between the highest occupied molecular orbital (HOMO) of a donor and the lowest unoccupied molecular orbital (LUMO) of an acceptor (12–14), there have been many OPV cells whose *V*_OC_ does not show such a dependence. In addition, although the *V*_OC_ is a physical quantity obtained under photoirradiation, the Δ*E*_HL_ has only been discussed under a dark condition so far. Thus, it is necessary to clarify the origins of *V*_OC_ for enlarging the *V*_OC_ of OPV cells.

Although the *η* of the OPV cells has been improved to more than 4% by forming a bulk hetero-junction (BHJ) that may increase the D/A interface area (4–6), BHJ is difficult to obtain D/A interface area reproducibly and to give a quantitative discussion between BHJ and *η*. Hence, BHJ certainly improves the *η* to some extent, but does not allow us to solve the two main issues described above.

The present paper therefore focuses on the following two basic aspects: 1) to clarify the influences of the structural properties (crystalline and molecular orientation) on the EQE, which plays a crucial role of determining *I*_SC_, by varying the crystal structure and/or molecular orientation of organic films in the OPV cells, because EQE depends directly on the diffusion length of excitons and/or carriers, and 2) to clarify the origins of open-circuit voltage *V*_OC_ by examining the correlations between *V*_OC_ and built-in potential (*V*_bi_) in the vicinity of D/A interface upon photoirradiation, because the *V*_bi_ of D and A films in the vicinity of the D/A interface is essentially affected by incident light wavelengths.

To achieve the basic aspects described above, we fabricated a simple D/A hetero-junction OPV cells. Zinc octaethylporphyrin [Zn(OEP)] and C_60_ were, respectively, used as a donor and an acceptor, because the former has a high photo absorption coefficient (15) and the latter has a high electron affinity (16). In addition, the strong interaction at the Zn(OEP) and C_60_ interface can be expected to generate carriers efficiently at the interface (17, 18).

## 1. Structural effects of Zn(OEP) films on the EQE of Zn(OEP)/C_60_ OPV cells

In this section, we examine how the structural properties of Zn(OEP) films affect EQE (19) by fabricating two kinds of OPV cells consisting of Zn(OEP) (20-nm- and crystalline 20-nm-thick) and 30-nm-thick C_60_ films on an ITO substrate (ITO/Zn(OEP)/C_60_/Al).

**Fig. 2:**
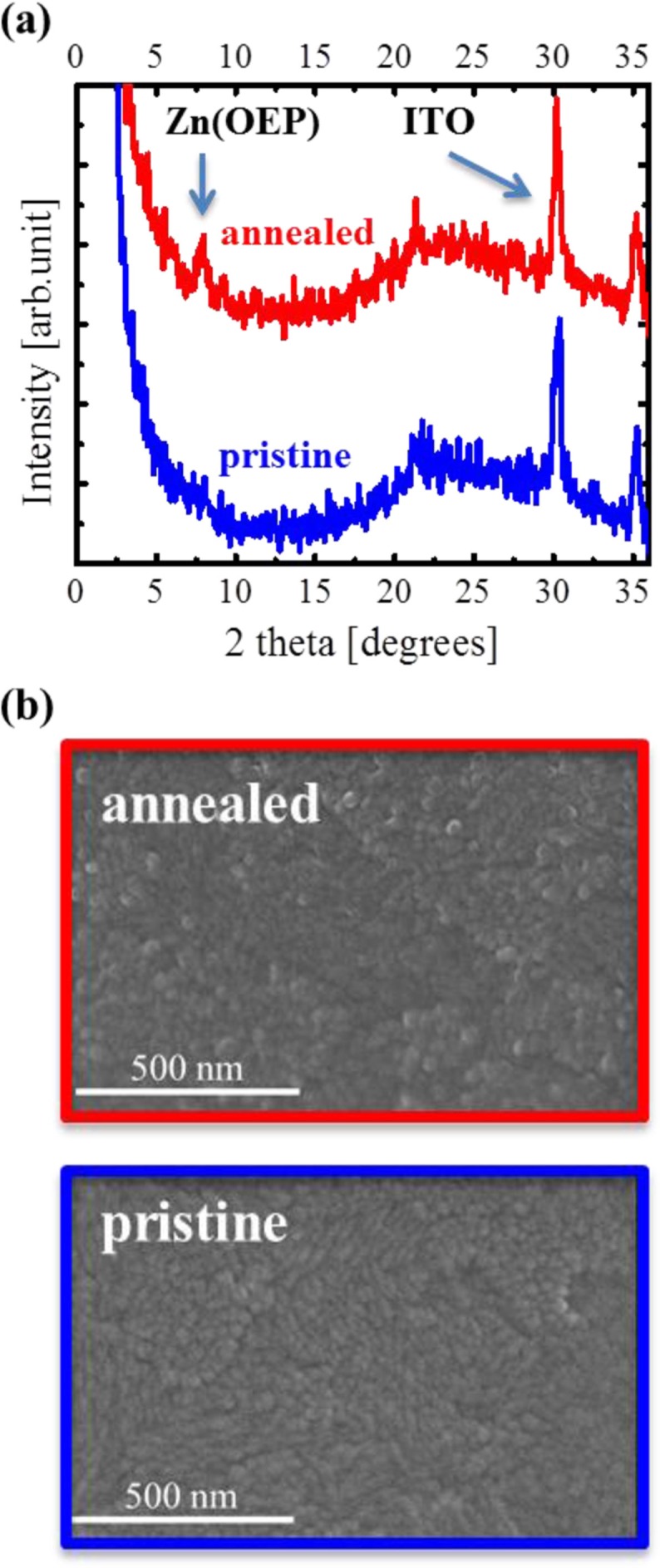
XRD patterns of pristine (blue) and annealed (red) 20-nm-thick Zn(OEP) films formed on an ITO (a) (20). SEM images of pristine (blue) and annealed (red) 20-nm-thick Zn(OEP) film formed on an ITO (b) (20).

We first investigated the crystal structure, molecular orientation, and surface/interface morphology of Zn(OEP) films on an ITO substrate spin-coated with and without the 3,4-polyethylenedioxythiophene:polystyrenesulfonate (PEDOT:PSS), by using x-ray diffraction (XRD) and scanning electron microscope (SEM), and succeeded in forming the crystalline Zn(OEP) films with no grain boundaries and a smooth surface morphology on the bare ITO substrate (20–22). PEDOT:PSS is generally used as a buffer layer between an ITO substrate and a donor layer, because PEDOT:PSS adjusts the work function at the anode for improving hole injection into the anode from a donor layer (23). PEDOT:PSS film also inhibits short circuits by planarization of the rough ITO surface (24). For Zn(OEP) films on an ITO substrate spin-coated with PEDOT:PSS, surface and cross-sectional SEM measurements found that Zn(OEP) molecules are significantly incorporated into the PEDOT:PSS film, and the Zn(OEP) films are not formed smoothly on the substrate. This indicates that PEDOT:PSS is unsuitable for the purposes of the present study, though it is widely used as a buffer material. Thus, a bare ITO substrate was used in the present work. We next examined a 20-nm-thick Zn(OEP) film formed on a bare ITO substrate at room temperature in an ultrahigh vacuum (UHV; a base pressure: 10^−7^ Pa), and found that the film is amorphous with a smooth roughness (see Fig. 2). When the 20-nm-thick amorphous Zn(OEP) film was post-annealed at 473 K for 1 min in UHV, it became a crystallized film including 20-nm-diameter grains, which was comparable to the film thickness, with smooth roughness as shown in Fig. 2 (the experiment details of XRD and SEM are described in Ref. 20). These facts demonstrate that the structural properties of Zn(OEP) films can be controlled on a bare ITO substrate.

We next examined how the structural properties of Zn(OEP) films affect the EQE of [ITO/Zn(OEP)/C_60_(30-nm-thick)/Al] OPV cells, by examining the amorphous 20-nm-, and crystalline (post-annealed) 20-nm-thick Zn(OEP) films, and discussed the structural effects on the EQE for the two kinds of the OPV cells, along with the photoabsorption efficiency and photoluminescence (PL) spectra of Zn(OEP) films (19).

### 1.1. Experiments

An ITO (120–160 nm thick) anode contact formed on glass, which is commercially available (Aldrich), was used as a substrate. A 1 mm-wide ITO contact was fabricated with a mask tape (Furukawa Electric) by using an etching solution (Kanto Chemical), the substrate was rinsed by acetone and thereafter by extra-pure water in an ultrasonic bath for 40 min, respectively. Subsequently, 20-nm-thick Zn(OEP) and 30-nm-thick C_60_ films were deposited on the 1 mm-wide ITO substrate at room temperature by thermal evaporation in an UHV chamber (a base pressure: 10^−7^ Pa). For the OPV cells consisting of the crystalline Zn(OEP) film, a deposited Zn(OEP) film was post-annealed at 473 K for 1 min before evaporation of C_60_. The ITO/Zn(OEP)/C_60_ sample was taken out of the UHV chamber and moved into the other vacuum chamber (a base pressure: 10^−5^ Pa). Thereafter, a 1 mm-wide aluminum (Al) cathode contact was deposited on the sample by thermal evaporation of an Al wire (Nirako, 99.999% pure) on a tungsten boat through a mask. Accordingly, the active area of the present OPV cells was 1 mm^2^.

The EQE was measured with an increment of 5 nm, using a lock-in amplifier (Toyo Corporation 5210) and a parallel monochromatic light (400–700 nm) modulated by a chopper with a frequency of 400–450 Hz. The monochromatic light was obtained from a 100 W halogen lamp (PHILIPS) and the white light through a monochromator and subsequently a bundle fiber. The monochromatic light from a bundle fiber became the parallel light with a diameter of approximately 7 mm through the objective lens. The reason why the parallel light was used in the present study is to prevent an experimental error of chromatic aberration. By adjusting the incidence angle of 90° normal to the samples under the optical microscope, only the device area of 1 mm^2^ was irradiated with the parallel monochromatic light passing through an iris.

### 1.2. Results and discussion

**Fig. 3:**
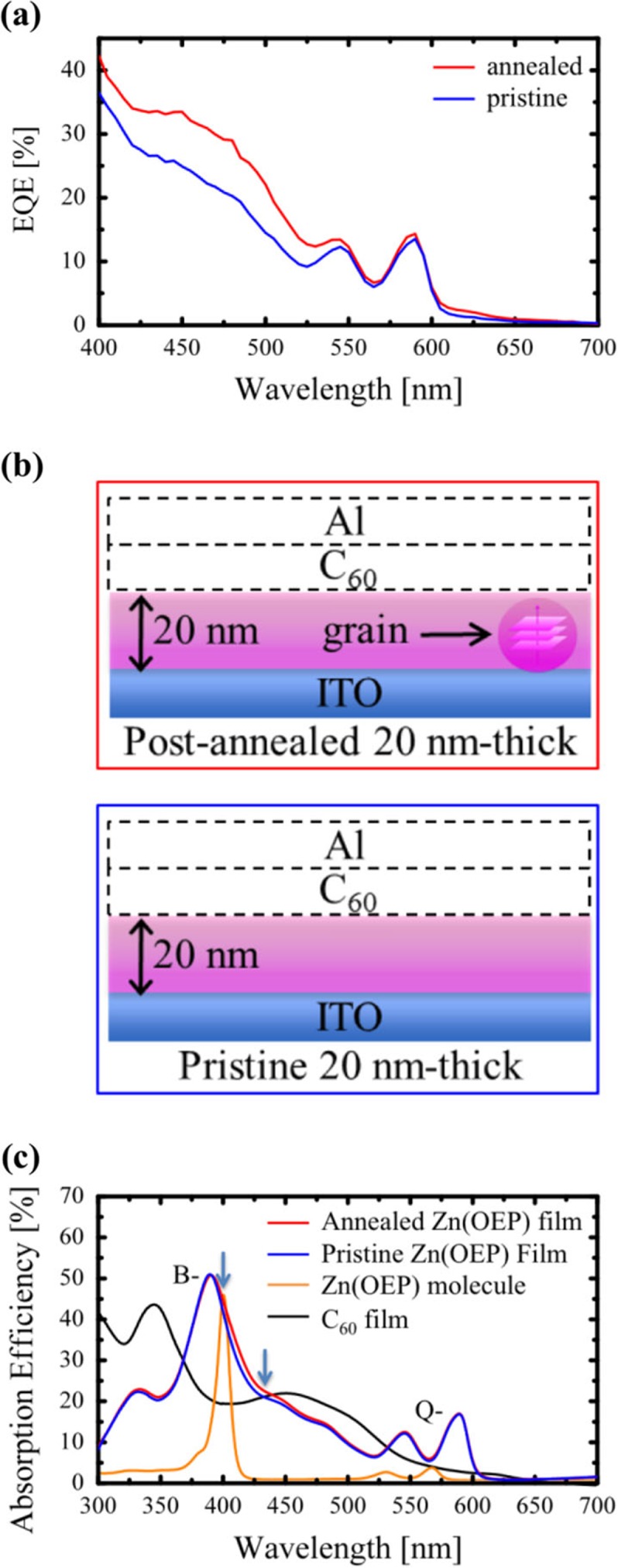
EQE of the OPV cells using pristine 20-nm- (blue) and post-annealed (crystalline) 20-nm-thick (red) Zn(OEP) films (a) (19). Schematic illustrations of the film structures for pristine and crystalline 20-nm-thick Zn(OEP) films (b) (19). The photoabsorption efficiency of pristine (blue), crystalline (red) 20-nm-thick Zn(OEP), and Zn(OEP) molecules in dichloromethane (orange), along with that of a 30-nm-thick C_60_ film (black) (c) (19).

Figure 3 shows: (a) the EQE spectra of the [ITO/Zn(OEP)/C_60_/Al] OPV cells using 20-nm (blue) and post-annealed (crystalline) 20-nm (red) thick Zn(OEP) films, (b) schematic illustration of the structure for each Zn(OEP) films, and (c) the photoabsorption efficiency of Zn(OEP) molecules in dichloromethane (orange), pristine (blue) and post-annealed (red) 20-nm-thick Zn(OEP) films, along with that of a 30-nm-thick C_60_ film (black). The OPV cell using the amorphous 20-nm-thick Zn(OEP) film exhibits the maximum EQE of 36% at 400 nm, which is comparable to that of previously reported OPV cells (35%) using buffer materials (25). Because it was confirmed that the surface morphology of each Zn(OEP) film used here remained almost unchanged by SEM (20), the C_60_/Zn(OEP) interfacial states and the contribution of excitons generated in the C_60_ film are the same as both the OPV cells, thus allowing us to discuss the structural effects of the Zn(OEP) films on the difference in EQE between them. As shown in Fig. 3a, the EQE increases entirely and the maximum EQE at 400 nm becomes greater from 36 to 42%, when the amorphous film is post-annealed. This may be due to the fact that the exciton diffusion length and/or carrier mobility becomes larger in the post-annealed crystalline Zn(OEP) film than in the amorphous one, because the grain size is comparable to the film thickness (there are almost no grain boundaries in the annealed film), as shown in Fig. 3b. In fact, in the range of 600–650 nm, although Zn(OEP) films exhibit no absorption band (see Fig. 3c), the EQE increases to some extent when the Zn(OEP) film is crystallized. This indicates that the carriers (holes) generated from excitons in the C_60_ film move in the crystalline Zn(OEP) film more effectively than in the amorphous one. Thus, the mobility of holes in the crystalline Zn(OEP) film is improved to some extent.

It is interesting to note that the EQE is particularly improved in the range of 420–520 nm compared to that of 550–600 nm. The EQE increases by 37% on average in this range, whereas the average increment in the other range is 16%. Photo-excitation of both B- (due to the second excited singlet state) and Q-bands (due to the first excited singlet state) shown in Fig. 3c result in the formation of intra-molecular excitons in Zn(OEP) films (26). Because the intra-molecular excitons are localized in the Zn(OEP) molecule, its diffusion length is not affected significantly by the structural properties (amorphous or crystalline) of the film. Thus, the EQE is not remarkably improved between the amorphous and crystalline films in the range of 520–630 nm. On the other hand, in the range of 420–520 nm for the photoabsorption efficiency of both Zn(OEP) films, isolated Zn(OEP) molecules have no corresponding bands, as shown in Fig. 3c. In the case of C_60_, the photoabsorption band of C_60_ films, which does not appear for isolated C_60_ molecules, was found to be due to inter-molecular charge-transfer (IMCT) excitons (27, 28). In a similar manner, the broad photoabsorption efficiency band of Zn(OEP) films in the range of 420–520 nm may arise from IMCT excitons formed between adjacent Zn(OEP) molecules. The evidence for existence of the IMCT excitons will be discussed later. As shown in Fig. 3c, the photoabsorption efficiency in the range becomes increased (1.06 times) slightly when the amorphous Zn(OEP) film changes to a crystalline one. This gives rise to an increase in the number of photo-generated IMCT excitons in the crystalline Zn(OEP) film, because a higher photoabsorption efficiency corresponds to a higher generation rate of IMCT excitons.

The EQE for OPV cells can be expressed by the product of the four efficiencies, EQE=*η*_A_ *η*_ED_ *η*_CG_ *η*_CT_, as described in the introduction. The present results found that the *η*_A_ and *η*_CT_ (hole) in the crystalline Zn(OEP) films slightly increase in the range of 420–520 nm and the whole range, respectively, and the *η*_CG_ could not be changed, because the D/A interfacial condition remained unchanged. Thus, these facts indicate that the *η*_ED_ enhances the EQE considerably in the range of 420–520 nm in addition to the *η*_A_ and *η*_CT_ for the OPV cells consisting of the crystalline Zn(OEP) film. The improvement of *η*_ED_ implies that the diffusion length of IMCT excitons increases in crystalline Zn(OEP) films, which play a key role of increasing the EQE for the present OPV cells.

**Fig. 4:**
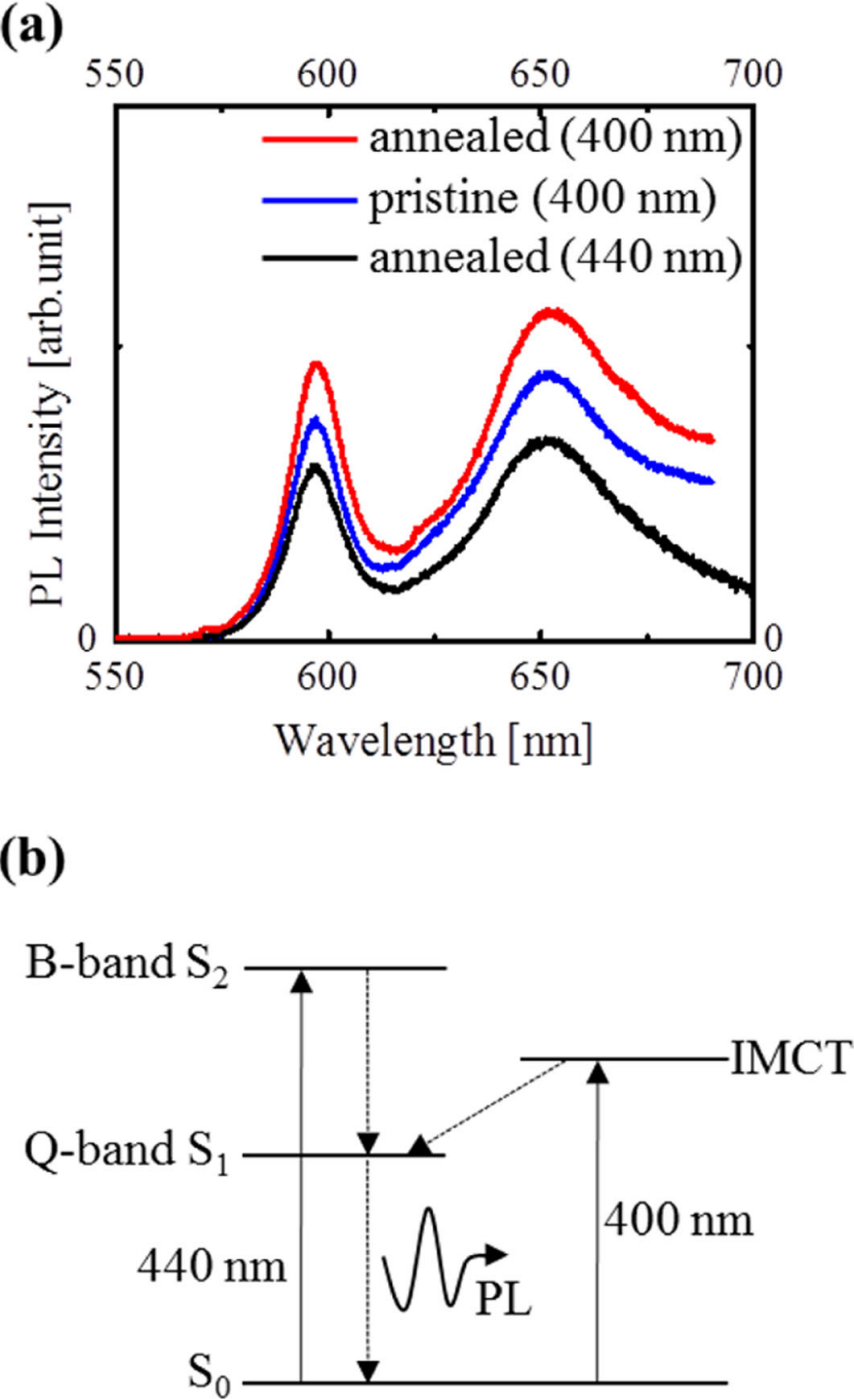
PL spectra of 20-nm-thick amorphous (blue) and crystalline (red) Zn(OEP) films at an excitation wavelength of 400 nm, and of 20-nm-thick crystalline Zn(OEP) films at that of 440 nm (black) (a) (19). The relaxation processes accompanied with the same PL in a similar manner to those for a C_60_ film (b) (19).

To confirm the generation of IMCT excitons in the amorphous and crystalline Zn(OEP) films, the PL spectra of the both films were measured by using two different excitation wavelengths of 400 and 440 nm (as indicated by the arrow in Fig. 3c) corresponding to the B-band and IMCT excitations, respectively. As shown in Fig. 4a, although the PL intensity excited by 440 nm was weaker than the others, the spectrum was observed in spite of the no-absorption band of the Zn(OEP) molecule itself. In addition, the spectral shapes were similar to each other and were attributed to the PL from the Q-bands (due to intra-molecular excitons). Such relaxation processes have been confirmed for PL spectra due to both the intra- and inter-molecular excitons for C_60_ films (27, 28). As shown in Fig. 4b, in the case of 400 nm light, electrons go up to the S_2_ state and immediately relaxed to the S_1_ state, and finally go down to the S_0_ state accompanied with the PL. On the other hand, in case of 440 nm light, electrons are excited to the IMCT state and subsequently return to the S_1_ state, and finally relaxed to the S_0_ state accompanied with the PL.

## 2. The origin of open-circuit voltage for Zn(OEP)/C_60_ hetero-junction OPV cells

In this section, in order to clarify the origins of *V*_OC_, we examined the correlation between open-circuit voltage *V*_OC_ and built-in potential *V*_bi_ in the vicinity of the D/A interface upon photoirradiation in Zn(OEP)/C_60_ hetero-junction OPV cells (29).

As noted in the introduction, since the determining factors of *V*_OC_ are still not understood, the crucial issue of a small *V*_OC_ (ca. 0.5 V) remains unsolved (30). Thus, it is important to elucidate the origins of *V*_OC_ and enlarge the *V*_OC_ for practical use of OPV cells. Much attention has been paid to understand the origins from the correlation between *V*_OC_ and the energy difference between the HOMO of the donor material and LUMO of the acceptor (Δ*E*_HL_) (10, 12–14). For some OPV cells, the *V*_OC_ was reported to be proportional to Δ*E*_HL_, and/or to be less than Δ*E*_HL_. On the contrary, some other OPV cells did not exhibit such a relationship (31–33). Although the origins of *V*_OC_ have been often discussed on the basis of the electronic structures at the D/A interface under a dark condition, the electronic states of organic films in the vicinity of their interface, such as built-in potential (*V*_bi_), under light irradiation were reported to be changed by photo-generated carriers (electrons and holes) (34, 35). For example, the surface potential of a dimethylquinquethiophene (DM5T) film on a Platinum (Pt) substrate decreases from 190 to 20 mV upon photoirradiation, because photo-generated holes are moved to the Pt substrate by a local electrical field at the interface (34). Thus, the electronic states at the D/A interface upon photoirradiation become important to clarify the origins of *V*_OC_.

The aim of this section is to reveal the correlation between *V*_OC_ and the *V*_bi_ in the vicinity of the D/A interface upon photoirradiation, using *in situ* impedance spectroscopy. The capacitance-voltage (*C–V*) characteristics obtained from impedance spectra can estimate the *V*_bi_ of each film in the vicinity of the D/A interface. We first examined the current-density versus voltage (*J–V*) characteristics of OPV cells consisting of 20-nm-thick Zn(OEP) and 30-nm-thick C_60_ layered films, respectively, connected with an ITO anode and Al cathode electrodes [ITO/Zn(OEP)/C_60_/Al] under monochromatic light (400, 440, 460, 485, 500, 545, and 590 nm) irradiation. Subsequently, the dependence of the short-circuit current-density (*J*_SC_), *V*_OC_, and FF on the wavelength of the monochromatic light was obtained. We finally discuss the difference in *V*_OC_ among the irradiation-light wavelengths in terms of *V*_bi_ and FF that are related to the accumulation and mobility of photo-generated carriers in both D and A films in the vicinity of their interface, respectively.

### 2.1. Experiments

The present OPV cells were fabricated under the condition described in Section 1.1. The *J*–*V* characteristics of the OPV cells were measured with a lock-in amplifier (Toyo Corporation, 5210) under irradiation of collimated monochromatic light (400, 440, 460, 485, 500, 545, and 590 nm) modulated by a chopper with a frequency of 400–450 Hz. The monochromatic light was obtained from a 100 W halogen lamp (PHILIPS) through a monochromator (JOBIN YVON, iHR320). The photon flux for each wavelength light was adjusted so that all were the same (65–95 nW/cm^2^). By taking an incident angle of 90° normal to the samples, only the active area of 1 mm^2^ for the present cells could be irradiated with the monochromatic light passing through an iris (1 mm in diameter).

Impedance spectra of the OPV cells were measured under dark and irradiation (400, 500, and 590 nm) conditions in the frequency range of 1–10^6^ Hz with respect to an external bias voltage in the range of −1.0–+1.0 V, using impedance spectroscopy (Solatron 1225B). This tool yields the impedance (*Z*) of each organic film in OPV cells non-destructively, based on the following equation:

3 $$Z=R_{\text{S}}+\frac{R_{\text{P}}}{1+\omega^{2}R_{\text{P}}^{2}C^{2}}-j\frac{\omega R_{\text{P}}^{2}C}{1+\omega^{2}R_{\text{P}}^{2}C^{2}}$$

**Fig. 5:**
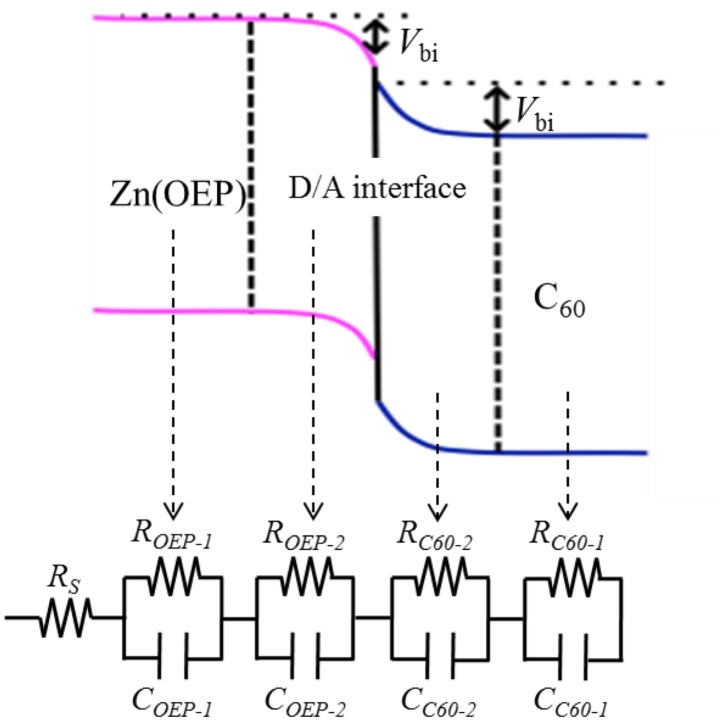
Equivalent circuit used for fitting the present impedance spectra, along with schematic illustration of the Zn(OEP)/C_60_ layered organic photovoltaic (OPV) cell (29).

Here, *R*_S_ and *R*_P_ denote the series and parallel resistances, respectively, *C* denotes the capacitance of each film, and *ω* is the frequency of the modulated applied-voltage. *R* and *C* were estimated by fitting the impedance spectra (so called Cole-Cole plots), in which x- and y-axes, respectively, denote real and imaginary parts of the impedance of OPV cells with a modified Eq. (3) based on the equivalent circuit shown in Fig. 5. In the present study, the software of Z-Plot (Solartron), which modifies Eq. (3) based on an equivalent circuit, was used for the fitting.

Figure 5 schematically illustrates the band energy model applied to the present OPV cells with the Zn(OEP)/C_60_ hetero-junction structure, along with its corresponding equivalent circuit consisting of a series resistance (*R*_S_), and four different resistances (*R*_OEP-1_ and *R*_OEP-2_, *R*_C60-2_ and *R*_C60-1_) and capacitances (*C*_OEP-1_ and *C*_OEP-2_, *C*_C60-2_ and *C*_C60-1_) for the Zn(OEP) and C_60_ films. As shown in Fig. 5, the resistance and capacitance of each film were separated into two different ones at locations far from and near to the D/A interface, respectively. Previous studies using Kelvin force microscopy and photoelectron spectroscopy indicated that band-bending is caused by charge carriers in organic films on any metal substrate and on organic films (36–39), and thus a band-bending model has often been applied to describe the D/A interfacial electronic structure for OPV cells (40). The typical size of the band-bending for organic films has been reported to be less than 15 nm (36, 37), which is smaller than the thickness of the present donor (20 nm thick) and acceptor (30 nm thick) films. In addition, since excitons generated in organic films are basically dissociated only at the D/A interface (namely, carriers generated only at the D/A interface), *V*_OC_ is predicted to be affected more significantly by the change in the electronic states in the vicinity of the D/A interface upon photoirradiation rather than by the organic/metal interface. Consequently, we employed the equivalent circuit shown in Fig. 5 on the basis of the band-bending in the vicinity of the D/A interface, and estimated the built-in potential (*V*_bi_) of the Zn(OEP) and C_60_ films near the D/A interface under dark and photoirradiation conditions.

We next briefly describe the procedure of evaluating *V*_bi_ from the impedance spectra of the OPV cells. For the 30-nm-thick C_60_ film, the capacitance of 1.23×10^−9^ F calculated from the reported relative permittivity value of 4.4 (41) was used as a fixed parameter for fitting the impedance spectra obtained under dark conditions, that is, (1/*C*_C60-1_)+(1/*C*_C60-2_)=1/(1.23×10^−9^ F). The reported relative permittivity was estimated from a C_60_ film in contact with an Al electrode, which is similar to the present OPV cells, and the capacitance calculated using the relative permittivity is equal to the series combination of the interface and geometrical capacitances (41). On the other hand, under photoirradiation, the *C*_OEP-1_ and *C*_C60-1_ values obtained under dark conditions were used as fixed parameters because the carriers are generated at the D/A interface as described above. When the impedance spectra obtained with respect to the bias voltage under photoirradiation conditions were fitted using the equivalent circuit shown in Fig. 5, each capacitance of Zn(OEP) and the C_60_ film was used as a fitting parameter. To estimate *V*_bi_ of each organic film in the vicinity of the D/A interface (OEP-2 and C60-2), the *C–V* characteristics of the OEP-2 and C60-2 films were examined using the following Mott–Schottky equation often used for organic films (42),

4 $$C^{-2}=\frac{2(V_{\text{bi}}-V)}{S^{2}q\varepsilon \varepsilon_{0}N}$$

Here, *V*, *q*, *ɛ*, *ɛ*_0_, *N, S*, and *C*, respectively, denote the external bias voltage in the range of −1.0–+1.0 V, elementary charge, relative dielectric constant, permittivity in vacuum, charge density, device active area, and capacitance obtained using the above-methods for each bias voltage. As seen in Eq. (4), when *C* ^−2^ is plotted as a function of *V*, *V*_bi_ can be evaluated by extrapolation of *C* ^−2^=0.

### 2.2. Results and discussion

**Fig. 6:**
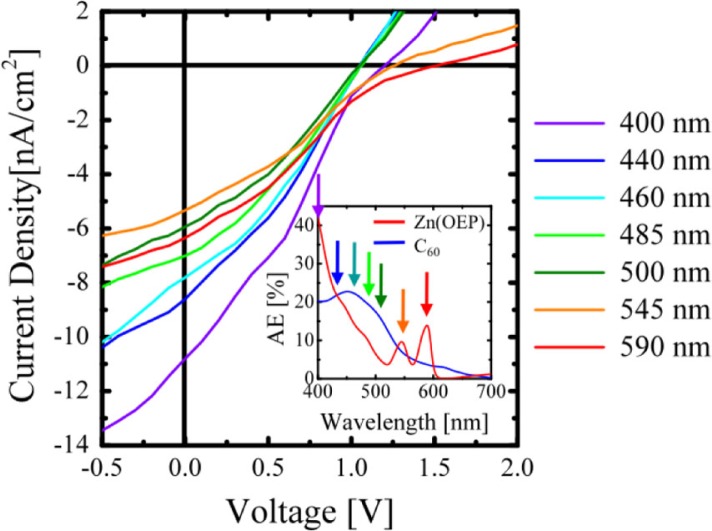
*J-V* characteristics of [ITO/20-nm-thick Zn(OEP)/30-nm-thick C_60_/Al] cells under irradiation with monochromatic light of 400, 440, 460, 485, 500, 545, and 590 nm wavelengths. The inset shows the absorption spectra of 20-nm-thick Zn(OEP) (red) and 30-nm-thick C_60_ films (blue) (29).

Figure 6 shows the *J–V* characteristics of the OPV cells under monochromatic photoirradiation with a wavelength of 400, 440, 460, 485, 500, 545, and 590 nm. The inset shows the photoabsorption efficiency (AE) of 20-nm-thick Zn(OEP) and 30-nm-thick C_60_ films. Table 1 summarizes the results of *J*_sc_, *V*_OC_, FF, and EQE obtained for each wavelength. The EQEs shown in Table 1 are quoted from the previous section. The *V*_OC_ was almost constant to be 1.05–1.06 V for 440, 460, 485, and 500 nm photoirradiations in which the C_60_ film absorbs photons remarkably, whereas it became larger to be 1.20, 1.26, and 1.51 V for 400, 545, and 590 nm photoirradiations in which photons are absorbed remarkably by the Zn(OEP) film, respectively. Since the dependence of the *V*_OC_ on the light intensity for 400 and 590 nm photoirradiations indicated that the *V*_OC_ is independent on the light intensity for both cases, the *V*_OC_ was found to depend upon the wavelength of light. In addition, *V*_OC_ in the range of 1.05–1.51 V is much larger than the Δ*E*_HL_ of 0.22 V estimated for the Zn(OEP)/C_60_ interface (3, 43). Accordingly, the determining factors of *V*_OC_ are strongly related to the electronic states of the OPV cells upon photoirradiation. To investigate the electronic states of the Zn(OEP) and C_60_ films near the D/A interface under photoirradiation, the impedance spectra of the OPV cells were next measured under dark and photoirradiation with wavelengths of 400, 500, and 590 nm, and subsequently the *C–V* characteristics of each film near the D/A interface were examined under each condition.

**Table 1:** Summary of *J*_SC_, *V*_OC_, FF and EQE of the [ITO/20-nm-thick Zn(OEP)/30-nm-thick C_60_/Al] cells under each irradiation condition (19)

Wavelength (nm)	*J*_SC_ (nA/cm^2^)	*V*_OC_ (V)	FF	EQE (%)
400	10.8	1.20	0.29	36.1
440	8.60	1.05	0.32	25.3
460	7.81	1.05	0.32	22.9
485	7.01	1.06	0.31	19.1
500	5.93	1.05	0.32	14.2
545	5.32	1.26	0.30	12
590	6.35	1.51	0.25	13.2

**Fig. 7:**
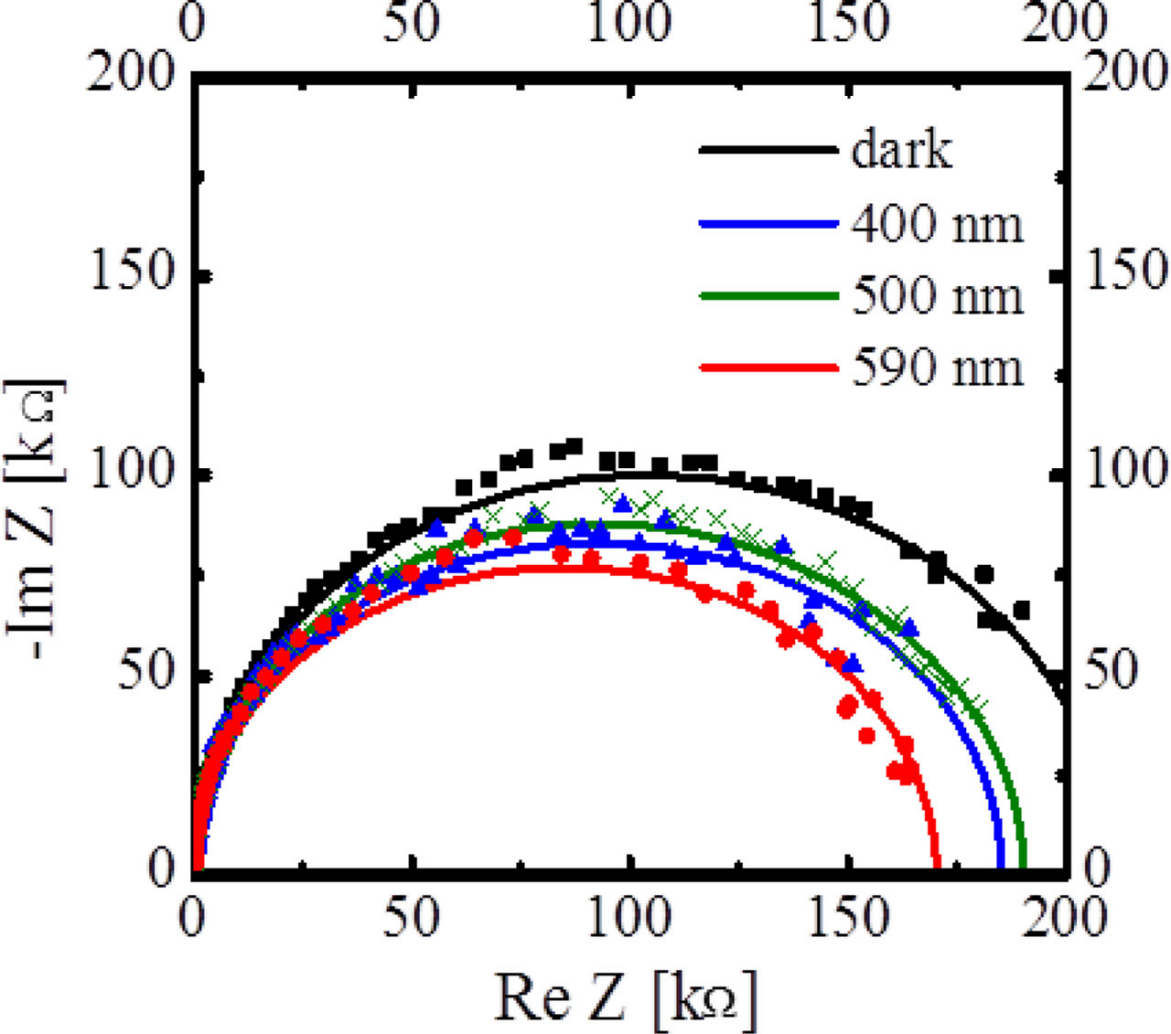
Impedance spectra of the OPV cell [ITO/Zn(OEP)/C_60_/Al] under dark (black), 400 (blue), 500 (green), and 590 (red) nm irradiation conditions (29).

Figure 7 shows the impedance spectra of the OPV cells before (dark) and under photoirradiation: dark (black), 400 nm (blue), 500 nm (green), and 590 nm (red). Here, the individual solid lines show the simulated results corresponding to the experimental results. Although two or more semicircles attributed to Zn(OEP) and C_60_ films and/or to their interfacial layers had been predicted to appear, only one-semicircle (precisely asymmetric shape) was observed for all the conditions. However, the Cole-Cole plots were not perfect semicircles, indicating that the results could not be fitted using an equivalent circuit consisting of a series resistance and one *RC* parallel circuit. This is because their time constants (=*R*×*C*) were almost the same order. In fact, when the thickness of Zn(OEP) and/or C_60_ films was varied separately, it was found that a part of the spectrum (black) in both low (less than ca. 1,500 Hz) and high (more than ca. 1,500 Hz) frequency regions is attributed to the C_60_ and Zn(OEP) films, respectively (44). As shown in Fig. 7, the impedance spectra change before and after photoirradiation and furthermore their changes depend on the wavelength of irradiation light. Thus, the capacitance of the organic films in the cell changes upon photoirradiation.

**Fig. 8:**
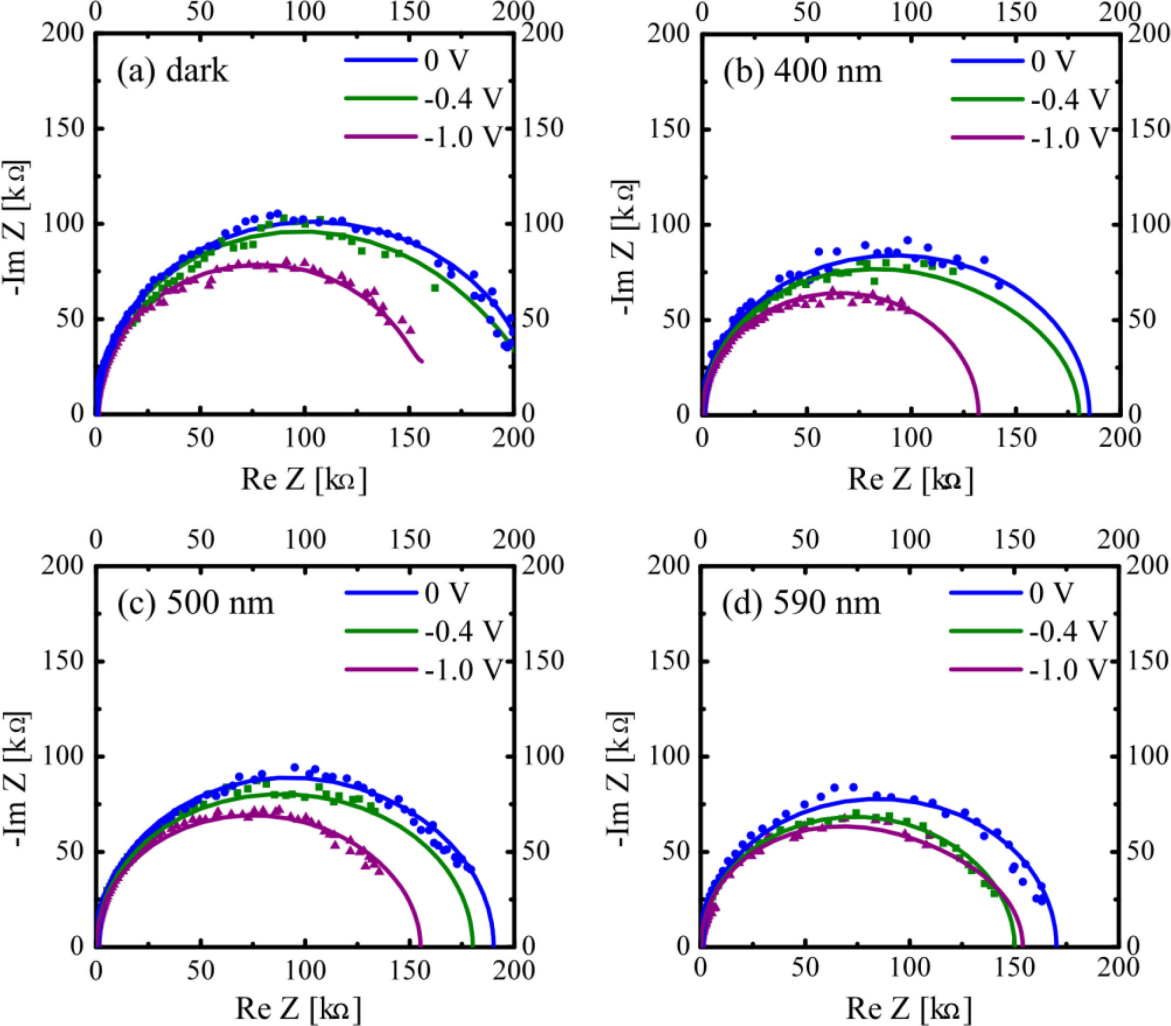
Dependence of impedance spectra of the OPV cell [ITO/Zn(OEP)/C_60_/Al] on applied voltage for dark (a), 400 nm (b), 500 nm (c), and 590 nm (d) irradiation conditions. Where, we chose typical impedance spectra obtained for 0 V (blue), −0.4 V (green), and −1.0 V (purple), though impedance spectra were obtained by varying the applied voltage from −1.0 V to +1.0 V (29).

To estimate the *V*_bi_ for each condition, the dependence of bias voltage (*V*) in the range of −1.0 V–+1.0 V was examined. Figure 8 shows the experimental (dot) and simulated (solid line) impedance spectra for each condition: *V*=0 V (blue circle), −0.4 V (green square), and −1.0 V (purple triangle) before (dark) and under 400 nm, 500 nm, and 590 nm photoirradiations. It was found that the shape of each impedance spectrum changed slightly by the applied voltage and their radius decreased with increasing the applied voltage. Such the results have been observed for bulk-hetero junction OPV cells (42). The *V*_bi_ of each film near the D/A interface was next estimated from the results of Fig. 8.

**Fig. 9:**
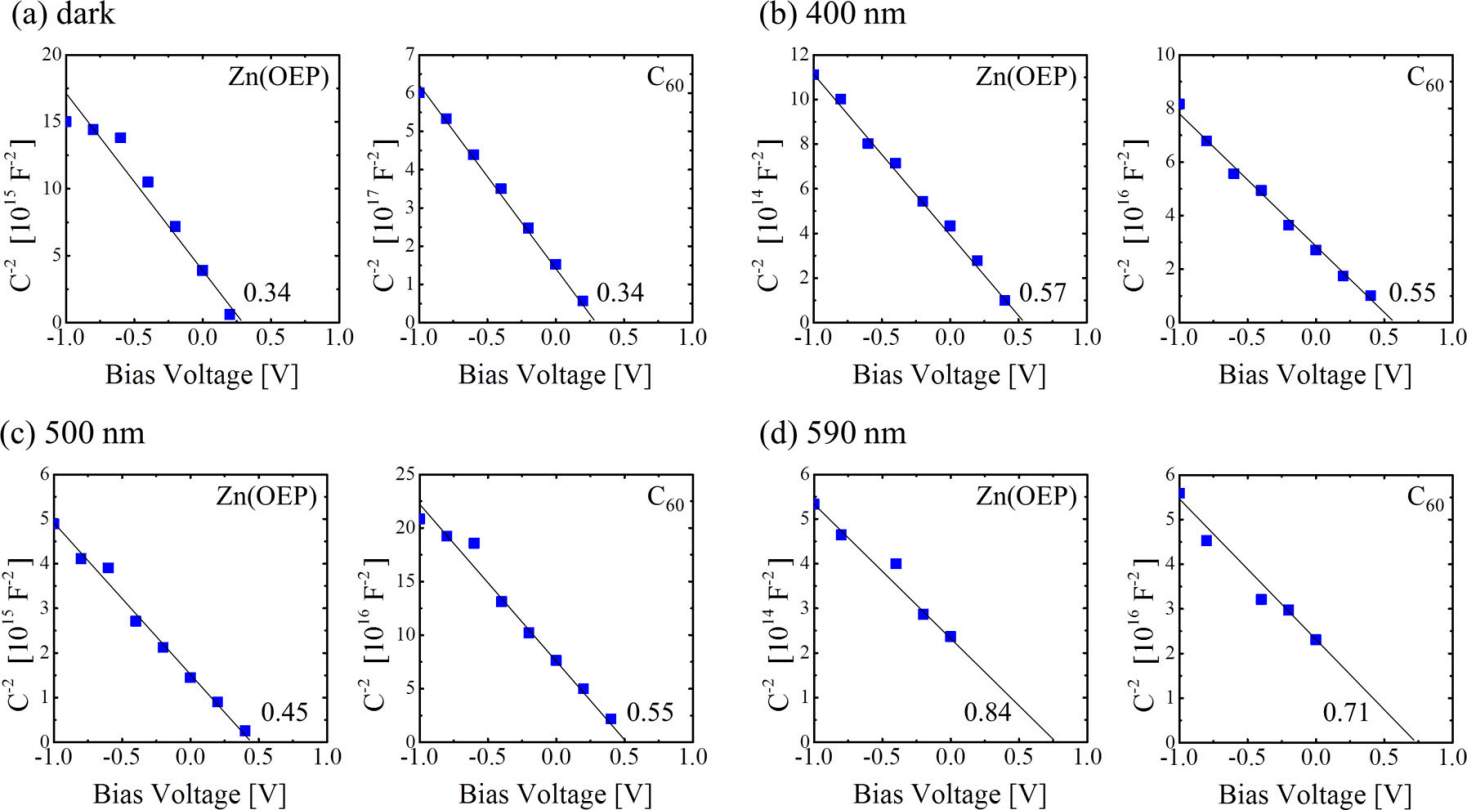
The *C* ^−2^ *–V* characteristics of Zn(OEP) and C_60_ films in the vicinity of the D/A interface for the OPV cell [ITO/Zn(OEP)/C_60_/Al] for dark (a), 400 nm (b), 500 nm (c), and 590 nm (d) irradiation conditions (29).

Figure 9 shows the *C* ^−2^–*V* characteristics of Zn(OEP) [OEP-2] and C_60_ [C60-2] films in the vicinity of the D/A interface under (a) dark, (b) 400 nm, (c) 500 nm and (d) 590 nm photoirradiation conditions. For the dark condition, the *V*_bi_ estimated from the *C* ^−2^–*V* characteristic results using Eq. (4) of the C60-2 film was obtained to be 0.34 V that is comparable to that of 0.30–0.45 V for a C_60_ film on metal (45), semiconductor (16), and organic films on an ITO substrate (37). This supports that the band-bending model shown in Fig. 5 is reasonable to be used for the present OPV cells. The *V*_bi_ of the OEP-2 film was the same value as for the C60-2 film. Conversely, when the OPV cells were irradiated with monochromatic light with wavelengths of 400, 500, and 590 nm, the *V*_bi_ increased upon photoirradiation in a similar manner to *V*_OC_ shown in Fig. 6, and showed the highest value of 0.84 and 0.71 V for Zn(OEP) and C_60_ films, respectively, under 590 nm photoirradiation. The increase in *V*_bi_ upon photoirradiation may be due to the accumulation of photo-generated electrons and holes in the vicinity of the interface (39), because a high resistivity of both organic films (for example, the resistivity of pristine C_60_ film is in the range of 10^8^–10^14^ Ωcm) probably prevents carriers (electrons and holes) from diffusing in the acceptor and donor films, respectively. Thus, *V*_bi_ should be strongly affected by carrier mobility, because the degree of accumulation of photocarriers increases with decreasing carrier mobility. On the other hand, since FF increases with carrier mobility (24), the wavelength dependence of *V*_bi_ is predicted to exhibit an opposite dependence to that of FF. We next examined the correlation among *V*_bi_, FF and *V*_OC_ obtained under each photoirradiation condition.

**Fig. 10:**
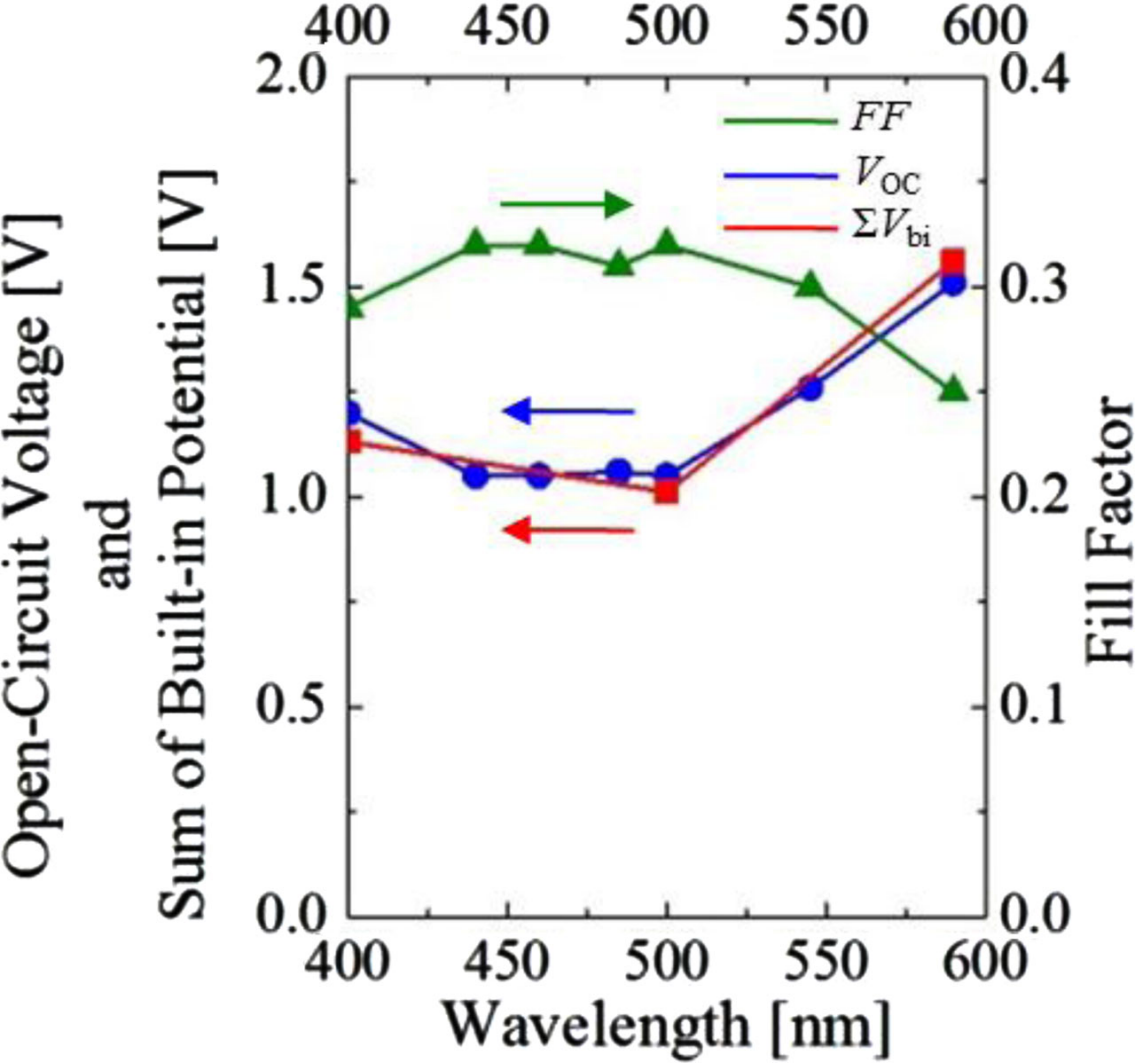
Plot of the open-circuit voltage *V*_OC_ (blue), the sum of *V*_bi_ (Σ*V*_bi_) (red), and the fill factor FF (green) as a function of irradiation-light wavelength (29).

Figure 10 shows a plot of the sum (Σ*V*_bi_) of *V*_bi_ (red-square) obtained for both Zn(OEP) [OEP-2] and C_60_ [C60-2] films, FF (green-triangle), and *V*_OC_ (blue-circle) as a function of monochromatic light wavelength. It is interesting to note that Σ*V*_bi_ is in good agreement with *V*_OC_. This implies that *V*_bi_ under photoirradiation plays a dominant role in determining *V*_OC_. Correspondingly, the wavelength-dependence of *V*_OC_ should also exhibit the opposite behavior to that of FF as well as *V*_bi_ because the dependence of *V*_bi_ is predicted to exhibit an opposite dependence to that of FF as described above. Indeed, the wavelength-dependence of both Σ*V*_bi_ and *V*_OC_ exhibits an opposite behavior to that of FF as seen in Fig. 10, which indicates that the accumulation of photo-generated carriers in the interfacial region increases *V*_bi_ and finally enlarges *V*_OC_. Such behavior of FF and *V*_OC_ with respect to monochromatic light resembles that of *J*_sc_, *V*_OC_, and FF with respect to temperature (33). FF (and *J*_sc_) and *V*_OC_ were, respectively, reported to increase and decrease with increasing carrier mobility (33). The correlations among Σ*V*_bi_, FF, and *V*_OC_ strongly suggest that accumulation of photo-generated carriers in the vicinity of the D/A interface plays a key role in determining *V*_OC_ for the present OPV cells.

According to the non-equilibrium thermodynamics theory (46), the potential energy (*E*) driving the diffusion of photo-generated electrons and holes is given as the sum of the electrical and chemical potential energies, that is,

5 $$\begin{array}{c} E_{\text{e}}=U_{\text{e}}+\mu_{\text{e}}\\ E_{\text{h}}=U_{\text{h}}+\mu_{\text{h}} \end{array}$$

Here, the *U* and *µ* denote the electrical and chemical potential energies, respectively, and the subscripts e and h denote electron and hole, respectively. The driving force for diffusion of photo-generated carriers can be expressed as ∇*E*, which is zero when the applied voltage *V* reaches *V*_OC_. Thus, ∇*U* corresponds to ∇(*V*_bi_ – *V*) used in the present study as follows:

6 $$\begin{array}{c} \nabla E=\nabla \left(V_{\text{bi}}-V\right)+\nabla \mu \\ 0=\nabla \left(V_{\text{bi}}-V_{\text{OC}}\right)+\nabla \mu \end{array}$$

Since ∇*µ* has been regarded as having a primary role in driving the photocarriers to each electrode rather than ∇*U* [∇(*V*_bi_–*V*)] for the OPV cells, the electrical potential *U* (*V*_bi_) in the D/A interfacial region has previously been considered to play a minor role in determining *V*_OC_ (46). However, as shown in Fig. 10, the electrical potential (*V*_bi_) in the D/A interfacial region upon photoirradiation plays a primary role in determining *V*_OC_ rather than *µ* and/or Δ*E*_HL_ for the solar cells in this work.

A quantitative discussion of the correlation between *V*_OC_ and band-bending in the vicinity of not only D/A but also metal/organic interfaces upon photoirradiation is future work for satisfactorily interpreting the origins of *V*_OC_ for OPV cells.

## 3. Summary

To investigate what the crucial factors are for improvement of the *η* of the OPV cells, we investigated the influence of Zn(OEP) film structures on the EQE and the origins of *V*_OC_, using OPV cells consisting of Zn(OEP) and C_60_ films [ITO/Zn(OEP)/C_60_/Al].

By using crystalline Zn(OEP) films with no grain boundaries, the maximum EQE obtained at 400 nm increased from 36 to 42%. Crystalline Zn(OEP) films played roles in both increasing the number of IMCT excitons by 1.06 times and by improving the carriers/IMCT excitons mobility in the Zn(OEP) films. Consequently, the EQE showed the most remarkable improvement of 1.37 times in the range of 420–520 nm, which corresponds to the Zn(OEP) photoabsorption band resulting in IMCT excitons. These results indicate that the improvement of the IMCT excitons mobility plays a key role in increasing the EQE, i.e. increasing the *I*_SC_ of the OPV cells. On the other hand, the *V*_bi_ of each film in the vicinity of the D/A interface was found to increase upon photoirradiation because a high resistivity of each organic film caused accumulation of photo-generated carriers in the D/A interfacial region. It was interesting to note that Σ*V*_bi_ is in good agreement with *V*_OC_, which suggests that the electrical field caused by charge accumulation of photo-generated carriers in the vicinity of the D/A interface plays a crucial role of determining *V*_OC_ of the OPV cells.

In these studies, the increase in IMCT excitons and accumulation of photo-generated carriers in the vicinity of the D/A interface are respectively found to be key factors in improving the *η* in terms of *I*_SC_ and *V*_OC_. Accordingly, 1) an improvement of the diffusion length of IMCT excitons by controlling film structures, and 2) a combination of donor and acceptor materials with both a low resistivity and a high *V*_bi_ are regarded as an essential approach to improve the energy conversion efficiency of OPV cells.
